# Effects of arbuscular mycorrhizal fungi and P-solubilizing Pseudomonas fluorescence (ATCC-17400) on morphological traits and mineral content of sesame

**DOI:** 10.1016/j.sjbs.2021.03.024

**Published:** 2021-03-17

**Authors:** Alpa Yadav, Ishan Saini, Prashant Kaushik, Mushtaq Ahmad Ansari, Mohammad Rashid Khan, Nazrul Haq

**Affiliations:** a Department of Botany, Indra Gandhi University, Meerpur, 122502 Rewari, India; b Department of Botany, Kurukshetra University Kurukshetra, Kurukshetra 136119, India; c Instituto de Conservación y Mejora de la Agrodiversidad Valenciana, Universitat Politècnica de València, Camino de Vera 14, Valencia 46022, Spain; d Department of Pharmacology and Toxicology, College of Pharmacy, King Saud University, Riyadh, Saudi Arabia; e Department of Pharmaceutics, College of Pharmacy King Saud University, Riyadh, Saudi Arabia

**Keywords:** *Acaulospora laevis*, *Glomus mosseae*, Microbes, Mycorrhizal fungi, *Pseudomonas fluorescens*, Soil

## Abstract

Sesame (*Sesamum indicum* L.) is an important staple crop of the family Pedaliaceae. The commercial production of sesame is still dependent on the applications of chemical fertilizers. Mycorrhiza inoculum resulted in better morphological and biochemical traits in vegetables. Thus, here the outcome of arbuscular mycorrhizal fungi (AMF) and *Pseudomonas fluorescence* (ATCC-17400) inoculation was studied in the pot culture experiment. Primarily, there seems to be a promising opportunity of AMF in sesame under pot and field trials because of enhanced morphological parameters, especially root weight, and disparities in nutrients and metabolites. The AMF appears to be an option to boost plant growth, mineral content, and sesame yield. The AMF treatment with *Pseudomonas fluorescence* strain (ATCC-17400) determined the maximum values for the morphological traits and mineral content. Overall, our study highlights mycorrhizal fungi and other microbes efficacy in achieving a successful sesame production.

## 1 Introduction

One of the most significant challenges of the 21st century is to feed the world competing with the increasing population rate. One of the economically important oil yielding plants is *Sesamum indicum* (Family: Pedaliaceae), commonly called Sesame. It is considered a vital oilseed source for developing countries as animal protein is costlier (Hassan et al. 2017). Sesame seeds have high oil content with a deliciously nutty aroma, mild taste and flavour, and biodiesel production quality (Habibullah et al. 2015). Sesame seeds contain a substantial amount of carbohydrates, proteins, fibres, essential minerals (Ca, P, Fe, etc.), tryptophan, methionine, lignans, flavonoids, phenolics, saponins and polyunsaturated fatty acids as well as rich in vitamin B and E (Myint et al. 2020).

Moreover, sesame seeds have several health benefits in terms of antioxidant, hypocholesterolemic effects, anticancer and blood pressure reduction activities, emulsifying, and foaming properties (Joshi et al., 2015, Hwang et al., 2017). Seeing the medicinal properties of sesame, some medicinal plants which may have active pharmaceutical compounds and other secondary metabolites are also in demand (Saini et al. 2020a). Thus, people are growing those plants in their home gardens, especially women in this way they are also conserving biodiversity (Saini et al. 2019a). This also increased the consumption of various wild and underutilized fruits with so many therapeutic properties (Cheema et al. 2016). Taking these properties in mind, the sesame plant is selected for the experiment.

Sesame is one of the first crop cultivated for oil production, and during the last 3 decades, the world production of sesame has noticeably increased (FAOSTAT, 2017). Global sesame seed consumption in 2018 was USD 6559.0 million, and it is estimated that sesame seed consumption will reach 7244.9 million by 2024. While in India, consumer preferences for sesame seeds have increased by 15% in the present time, occupying an area of 1.8 M hectares (Bheda, 2019). To meet the increasing demand of consumers, there should be continuous production of food crops and oilseed crops. Thus, growers use non-renewable inorganic fertilizers in an excessive dose that sometimes creates a problem in the soil and hinders rhizospheric interaction of roots and soil microbes (Saini et al. 2019a). Therefore, a better approach is to choose a biologically active, sustainable, and friendly method. Such a process can be the use of microbes as bio-inoculants, especially Mycorrhizal Fungi (earlier known as Vesicular Arbuscular Fungi) and Phosphate Solubilizing Bacteria (PSB), also known as Plant Growth Promoting Rhizobacteria (PGPR) (Azooz and Ahmad, 2013, Hashem et al., 2019).

Arbuscular Mycorrhizal Fungi (AMF) are obligate symbiont that is thought to be largely promiscuously, colonizing more than 95% of vascular plants (Graham et al. 2017). AM fungi have a mutual relationship between plants receiving carbon source (lipid + carbohydrate) from the plants and contribute an adequate amount of water and minerals to the plants (Chen et al. 2018). During colonization, AMF produces lipo-chito-oligosaccharides perceived by the plant roots through their signalling molecules (strigolactones and cutin monomers) that activate a typical symbiosis signalling cascade (Bonfante and Genre, 2015, Konieczny and Kowalska, 2017). AMF interaction improves nutritional status, manages the soil microenvironment, restores degraded land, and reduces nutrient leaching (Cavagnaro et al. 2015). PSB (*Pseudomonas fluorescens*), on the hand, help AMF and vice versa. However, agriculture soil receives a large number of fertilizers, mainly NPK and lacks an excellent nutrient retention system, but AMF/PSB inoculation can manage soil nutrient recycling. (Cavagnaro et al. 2015). Application of AMF and PSB benefit crop development by enhancing plant health and yield (Rouphael et al. 2015). AMF and PSB's main role is absorbing and translocating mineral nutrients (particularly P) beyond the depletion zone and induce nutraceutical values as well as interfere with the plant growth hormones, thereby influencing better growth along with inducing tolerance to various environmental stresses (Rouphael et al. 2015). PSB and AMF activate the plant’s defense mechanism against pathogen attack (called systemic acquired resistance), which is very well documented (Lanfranco et al., 2018, Saini et al., 2020c). *P*. *fluorescens* is gram-negative, rod-shaped PGPR that colonize plant roots by secreting greenish fluorescent pigment (hence named) particularly under iron scarcity (Kalita and Ram, 2019). Collectively, *P*. *fluorescens* helps in dissolving the complex inorganic phosphate in the soil and mycorrhizal fungi absorb them and transfer to plants (Anokhina et al., 2018).

While other researchers found AMF to be beneficial for the plants and found, how they are interacted with other soil microbes via large scale sequencing or metagenomics studies (Spatafora et al., 2016, Kaushik et al., 2020). Researchers are utilizing biotechnological approaches in the quest for biofortified crops. But, soil amendments using AMF are more ecofriendly and useful (Malhi et al. 2021). Owing to the importance of Sesame with easy compatibility of *Pseudomonas fluorescens* (PSB), AM fungi – *Glomus mosseae* (=*Funneliformis mosseae*), and *Acaulospora laevis,* the present investigation was planned for enhancing the yield of sesame, under pot conditions.

## 2 Materials and methods

### 2.1 Study site, soil characterization and pot preparation

Experiment was designed under a controlled temperature of 22 ± 2 °C, 16-h photoperiod light of 8000 lx (in addition to natural sunlight) and 65–70% humid condition in polyhouse of Botany Department, Kurukshetra University, Kurukshetra, during 2018–2019. Soil, taken from the Botanical garden of Kurukshetra University, was first air-dried, sieved through 2 mm sieve, autoclaved to eliminate previous microbial strains, and then mixed with sand in 1:3. Soil pH was 7.5 measured by pH meter (PHS-3C, Shanghai Lida Instrument Factory). Other characters of soil are as follows- sand: 71.05%, clay: 5.24%, silt 3.65%, Ca: 0.76%, K: 4.97: Mg: 0.81%, P: 0.57%, Fe: 12.95%, Zn: 8.77% (Estefan et al., 2013a, Estefan et al., 2013b). The seeds of Sesame variety RT46 were procured from Oil Seed Section of CCS Haryana Agricultural University, Hisar, Haryana 125,004 (India). This variety was chosen because of its cultivation at an extensive scale and its nutritional profile and disease resistance. Later were cleaned with 0.5% (v/v) sodium hypochlorite for 10 min, after washing with water were sown in each pot.

The experimentation was carried out in earthenware pots (24.5 × 24), using a randomized complete block design (RCBD) with five replicates of each treatment. To each pot sterilized sand: soil mixture inoculum was added. 1 kg full in control pot and 100 g less in other treatment as AMF soil inocula were applied.

### 2.2 Procurement and mass multiplication of AMF and Pseudomonas fluorescens

Inoculum of *Glomus mosseae* containing 75–78% colonization (root pieces) plus 670–690 AM spores (w/w) and *Acaulospora laevis* containing 68–71% colonization (root pieces) plus 550–570 AM spores (w/w) were procured from Forest Pathology Discipline, Forest Protection Division, FRI, Dehradun. Both inoculums were then mass multiplied using Barley (host) for almost 3 months (as AMF generally take 80–90 days for infection and full colonization) to develop the starter inocula for the experiment (Saini et al. 2020c). After mass production, starter inocula was ready containing *G*. *mosseae* with 72–74% colonization/infection (barley root pieces) plus 620–640 spores (w/w) and *A*. *laevis* with 64–67% colonization/infection (barley root pieces) plus 540–560 (w/w) [quantified by gridline intersect method by Adholeya and Gaur (1994); estimation of root colonization percentage was done by Philips and Hayman (1970)]. *Pseudomonas fluorescens* (ATCC-17400) was obtained from the CSIR-Institute of Microbial Technology (CSIR-IMtech), Chandigarh, India. It was then multiplied in a nutrient broth medium containing beef extract: 3 g/L; peptone: 5 g/L, and NaCl: 5 g/L, respectively to develop the bacterial colonies. The medium was then incubated at 32 °C for 48 h for proper bacteria growth.

### 2.3 Noculation of AM fungi and Pseudomonas fluorescens

For single AMF treatment, 100 g of each inoculum was supplemented per pot and 50 g of each inoculum for dual and consortium treatments (Saini et al. 2020b). For *P*. *fluorescens* treatment, all the seeds were dipped in the nutrient broth medium for 10 min

The experiment had seven treatments, as follows:

1. Control (without any bioinoculant)
2. *Glomus mosseae* (G)*
3. *Acaulospora laevis* (A)
4. *Pseudomonas fluorescens* (P)
5. *G*. *mosseae* + *A*. *laevis* (G + A)
6. *G*. *mosseae* + *P*. *fluorescens* (A + P)
7. *A*. *laevis* + *P*. *fluorescens* (A + P)
8. *G*. *mosseae* + *A. laevis* + *P*. *fluorescens* (G + A + P)

* *Glomus mosseae* is now known as *Funneliformis mosseae*.

Hoagland’s nutrient solution without P (100 ml/pot) was applied after every 15th day of transplantation. Plants were watered properly time to time.

### 2.4 Plant harvest and analysis

Plants were harvested after 120 days of inoculation (DOI), and the effect of bioinoculants on various growth parameters were reported.

### 2.5 Change in plant height and root length

Plant height (cm) was measured after 120 DOI by 1-meter scale, and roots were also measured after uprooting.

### 2.6 Root and shoot biomass

After plants were harvested by carefully uprooting and gently washed under running water to remove the adhering soil. Root and shoot dry weight were recorded separately by keeping them in an oven overnight at 70 °C.

### 2.7 Oil extraction

The sesame seed oil estimation was done by petroleum ether using the Soxhlet’s procedure (AOCS, 1997) with a boiling range between 400 and 600°C. 300 ml of normal Hexane was taken, and 10 g of the test was positioned in the thimble and placed in the extractor's centre. The Soxhlet was warmed up at 60oC. If the solvent was boiling, the vapour increases from the vertical tube to the pinnacle's condenser. The extract seeps through the thimble's skin pores and fills the siphon tube, wherever it flows back down into the round outsole flask. This was permitted to carry on for thirty minutes. It was then taken from the hose, dried out in the oven, cooled in the desiccators, and weighed once again to identify the quantity of oil extracted. After the removal, the ensuing combination (miscella) that contains the oil was warmed up to recuperate solvent in the engine oil. 5 replicates of every treatment were taken.

### 2.8 Protein analysis

Protein was estimated by Bradford's (1976) method using Coomassie Brilliant Blue G-250 dye. 100 mg of sample was warmed with 10 ml 80% ethanol on a water bath for 2–5 min and allowed to cool down at room temperature. After this, the mixture was homogenized with the same ethanol in pestle and mortar, centrifuged at 5000 rpm for 10 min. After discarding the supernatant, the residue was re-extracted with 5 ml of 1 N NaOH and dissolved by keeping in the water at a temperature of 40–50 °C for 20–30 min. After 30 min, it was centrifuged again at 5000 rpm for 20–30 min, and the absorbance was recorded at 595 nm using a UV–vis spectrophotometer (Specord-205 Analytic Jena, Germany).

## 3 Nutrient uptake examination

P was determined by ammonium vanadate-ammonium-molybdate yellow colorimetric method for macronutrient analysis using a spectrophotometer (Model U-5100, Hitachi Co., Tokyo, Japan) that measured at 420 nm (Estefan et al., 2013a, Estefan et al., 2013b). Other macronutrients K, Ca, and Mg were determined by atomic absorption spectrophotometry (Model Z-2300, Hitachi Co., Tokyo, Japan) explained by Mitchell et al., 1974.

Seed samples were taken separately for micronutrient (Fe and Zn). Seed samples of 0.4 g were digested by 5 ml conc. HNO_3_ and 2 ml of conc. H_2_SO_4_ in a closed microwave digestion system (MARSxpress, CEM Corp.). Fe and Zn then analyzed with an inductively coupled plasma optical emission spectrometer (ICP-OES; Vista-Pro Axial; Varian Pty Ltd., Australia) explained by Kurt (2018).

### 3.1 Statistical analysis

Statistical interpretation of the data was performed using one-way analysis of variance (ANOVA) followed by post hoc test through computer software SPSS 16.0 (SPSS Inc.Chicago, IL, USA). Duncan’s Multiple Range Test (DMRT) was used for comparison and mean ranking at P < 0.05 level of significance.

## 4 Results

**Fig. 1:**
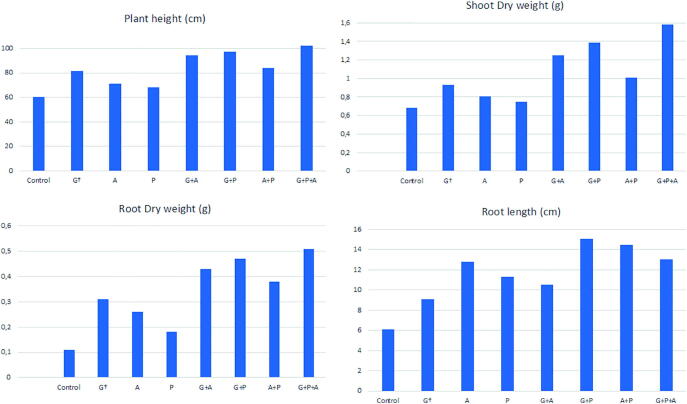
Variation among the 8 different of sesame for plant height (cm), shoot dry weight (gm), root dry weight (gm) and root length (cm).

It is evident from Fig. 1 that inoculated or treated plants showed a significant increase in growth compared to the control. Data on change in plant height was substantial in all the treated plants, and maximum plant height was observed in the mixed consortium of *G. mosseae* + *A. laevis* + *P. fluorescens* (102.18 ± 2.56) followed by dual inoculation of *G. mosseae* + *P. fluorescens* (97.13 ± 2.13) treated plants, which was far better than the uninoculated control plants (50.24 ± 3.88). Maximum root length increment was also observed in the consortium of *G. mosseae* + *A. laevis* + *P. fluorescens* (13.03 ± 0.27) and least in control (6.11 ± 0.27). It is evident from Fig. 1 that dry weight of all the inoculated Sesame plants were found to be significant in terms of shoot and root. It was found that supreme increment in shoot dry weight was recorded in the combination of *G. mosseae* + *A. laevis* + *P. fluorescens* (1.58 ± 0.63) followed by *G. mosseae* + *P. fluorescens* (1.25 ± 0.53) (Fig. 1). Similarly, the increase in root dry weight was also observed to be maximum with *G. mosseae* + *A. laevis* + *P. fluorescens* (0.47 ± 0.34) followed by *G. mosseae* + *P. fluorescens* (0.51 ± 0.43) (Fig. 1).

Based on this investigation, it seems that mycorrhiza's application with other bioinoculants (*P. fluorescens*) did have much constructive effect on AM spore number and mycorrhizal root colonization. Maximum increment in percent mycorrhizal root colonization was observed in the consortium treatment, i.e., *G. mosseae*, *A. laevis* and *P. fluorescens* (98.41 ± 1.14) followed by the combination *G. mosseae* + *P. fluorescens* (74.51 ± 3.83). AM spores’ number being highest in mycorrhizospheric plants treated with *G. mosseae* + *P. fluorescens* (174.38 ± 3.16) followed by a mixture of *G*. *mosseae* + *P. fluorescens* (157.42 ± 2.92). AM root colonization and AM spores were not observed in *P*. *fluorescens* and control treatments*.*

It is apparent from Table 1 that percent of seed protein and oil content varied among different treatments. Maximum protein and oil content was recorded in the consortium of *G. mosseae* + *A. laevis* + *P. fluorescens* (Protein: 21.08 ± 0.87; Oil: 46.30 ± 0.64) followed by *G. mosseae* + *P. fluorescens* (Protein: 19.67 ± 0.64; Oil: 43.95 ± 0.95) as compared to control (Protein: 17.11 ± 0.68; Oil: 38.58 ± 0.33). Data in Table 2 revealed that P, Mg, K, Ca, Fe, and Zn uptake was significantly affected by AM fungi and *P. fluorescens* in seeds. The highest value of P was recorded in *G. mosseae* + *A. laevis* + *P. fluorescens* (1.33 ± 0.79), followed by *G. mosseae* + *P. fluorescens* (1.19 ± 0.77) over control (0.65 ± 0.56). Likewise, other nutrients were also found maximum in the same consortium treatment (K: 7.91 ± 0.65; Ca: 1.88 ± 0.71; Mg: 1.81 ± 0.54; Fe: 16.51 ± 0.39; Z: 12.11 ± 0.77) as compared to control one (K: 5.88 ± 0.73; Ca: 0.87 ± 0.58; Mg: 0.86 ± 0.55; Fe: 13.02 ± 0.34; Z: 9.39 ± 0.45).

**Table 1:** Influence of AM fungi and *Pseudomonas fluorescence* on growth and yield parameters of Sesame.

Treatments	Root colonization (%)	AM spore number/10 g soil	Protein content (%)	Oil content (%)
Control	0 + 0^f^	0 + 0 ^g^	17.11 ± 0.68^e^	38.58 ± 0.33^f^
G†	61.39 ± 3.29^de^	111.22 ± 1.58^e^	18.11 ± 1.52 ^cd^	41.08 ± 0.75^c^
A	48.91 ± 3.21^e^	78.33 ± 5.38^f^	18.31 ± 1.51^d^	39.73 ± 0.66^de^
P	0 + 0^f^	0 + 0 ^g^	17.68 ± 0.42^e^	39.18 ± 0.71^e^
G + A	74.51 ± 3.83^e^	132.55 ± 1.58^c^	19.34 ± 1.57^b^	42.08 ± 0.75^bc^
G + P	87.53 ± 1.25^bc^	157.42 ± 2.92^b^	19.67 ± 0.64^b^	43.95 ± 0.95^bc^
A + P	63.31 ± 1.67^d^	120.74 ± 2.24^d^	18.76 ± 1.58^c^	41.87 ± 0.74^c^
G + P + A	98.41 ± 1.14^a^	174.38 ± 3.16^a^	21.08 ± 0.87^a^	46.30 ± 0.64^a^
L.S.D(P ≤ 0.05)	6.622	12.618	3.505	2.331
ANOVA(F 7,16)	386.297	372.955	3.571	72.859

†G: *Glomus mosseae* (=*Funneliformis mosseae*), A: *Acaulospora laevis*, P: *Pseudomonas fluorescens,* ±: Standard deviation.

‡Mean value followed by different alphabet/s within a column do not differ significantly over one other at *P* ≤ 0.05 (Duncan’s Multiple Range Test).

**Table 2:** Influence of AM fungi and *Pseudomonas fluorescence* on the mineral content of sesame seed.

Treatments	Calcium g 100 g^−1^ FW	Potassium g 100 g^−1^ FW	Magnesium g 100 g^−1^ FW	Phosphorus g 100 g^−1^ FW	Iron mg g^−1^ FW	Zinc mg g^−1^ FW
Control	0.87 ± 0.58 ^h^‡	5.88 ± 0.73^f^	0.86 ± 0.55^f^	0.65 ± 0.56 ^g^	13.02 ± 0.34^f^	9.39 ± 0.45^e^
G†	1.05 ± 0.39^e^	6.87 ± 0.92^b^	1.11 ± 0.81^de^	0.89 ± 0.45^e^	15.07 ± 0.22^c^	10.75 ± 0.78 ^cd^
A	1.08 ± 0.55^d^	6.08 ± 0.64^d^	1.36 ± 0.53^d^	0.75 ± 0.58^e^	14.25 ± 0.21^d^	10.53 ± 0.79 ^cd^
P	0.94 ± 0.72 ^fg^	5.35 ± 0.57^e^	0.95 ± 0.68^e^	0.71 ± 0.68^f^	13.87 ± 0.23^e^	10.19 ± 0.56^c^
G + A	1.69 ± 0.52^bc^	6.46 ± 0.36^c^	1.58 ± 0.47^bc^	1.08 ± 0.56^c^	16.01 ± 0.52^ab^	11.97 ± 0.65^b^
G + P	1.48 ± 0.38^b^	7.69 ± 0.73^a^	1.67 ± 0.62^ab^	1.19 ± 0.77^b^	15.94 ± 0.63^b^	11.75 ± 0.62^b^
A + P	1.36 ± 0.39^c^	7.08 ± 0.61^ab^	1.51 ± 0.29^bc^	0.97 ± 0.53^de^	15.64 ± 0.63^b^	11.07 ± 0.51^bc^
G + P + A	1.88 ± 0.71^a^	7.91 ± 0.65^a^	1.81 ± 0.54^a^	1.33 ± 0.79^a^	16.51 ± 0.39^a^	12.11 ± 0.77^a^
L.S.D(P ≤ 0.05)	1.401	0.185	0.218	1.811	0.129	1.721
ANOVA (F 7,16)	113.458	172.849	165.556	211.213	122.901	181.231

†G: *Glomus mosseae* (=*Funneliformis mosseae*), A: *Acaulospora laevis*, P: *Pseudomonas fluorescens,* ±: Standard deviation.

‡Mean value followed by different alphabet/s within a column do not differ significantly over one other at *P* ≤ 0.05 (Duncan’s Multiple Range Test).

## 5 Discussion

In order to determine the effect of microbial inoculation, it is very evident to know the feasibility of those microbes, particularly their combine outcome as different microbes have a different degree of colonization (Ambrosini et al. 2016). The present investigation showed that microbial inoculated sesame plants grew well with the different combinations used compared to control plants. It is apparent from the results that consortium treatment having AMF and *P*. *fluorescens* was proved to be the best treatment. AM fungi are involved in stimulating strigolactones responsible for improving root and shoot growth, which might be why our study sesame plant height and root-shoot dry weight ratio increased compared to control (Liu et al., 2018). The hyphopodia of AMF enter the plant root cortex to obtain lipid plus carbohydrate from the host plant (Sesame) and help the roots with increase uptake of nutrients, significantly P, which is compulsory for nucleic acids, enhancing the vegetative growth (Bona et al. 2017). This might be the reason for the higher plant height and plant weight of sesame with PSB and AMF inoculation. Treatment/s comprises of *A*. *laevis* and *G*. *mosseae* showed diverse results in all parameters studied because the extent of sap (water + minerals) absorption might differ due to different colonization (Wang and Jiang, 2015). Similarly, these AM fungi, when combining with *P*. *fluorescens,* possess a different absorption rate (Rashid et al. 2016). AMF produce organo-polysaccharides exudates that are decisive for soil porosity and quality, additionally when AMF inoculants mixed with *P*. *fluorescens* activity of theses exudates become more commendable (Rashid et al. 2016).

*P*. *fluorescens* and AMF secrete some phosphatase enzymes that solubilize orthophosphates, by this plant’s roots get easy access of not only P but other nutrients also like Mg, N, Zn, Cu, Fe, Ca, Se, etc. (Saini et al., 2019b, Johri et al., 2015). That’s why in our experiment nutrient level is increased. AMF and PSB increase the β-glucosidase, hydrolase and urease activities that may lead to good microbial growth in the rhizosphere, consequently maintaining the soil micro-environment and help in growth (Song et al. 2015).

The amount of different minerals absorbed directly affects phytohormones, which also get activated (Park et al. 2017). This increased in plant growth hormones and nutrients enhance the protein and oil content in our sesame plant. Resultantly, AMF and PGPR application enhanced the mineral nutrition absorption leading to enhanced photosynthesis (Arif et al. 2016). As discussed, there is an increase in nutrient accumulation in plants *via* AMF, one such nutrient, i.e., Nitrogen also absorbed faster, which is very beneficial for plants' protein formation (Balestrini et al. 2018).

Additionally, it is reported that there is up-regulation of some aquaporin genes, nitrate transporters and plant glutathione-S-transferases by applying AMF and PSB, which facilitate and maintain ionic/water balance in the plants even under drought stress (Jia-Dong et al. 2019). The increment of *G. mosseae* and *A. laevis* in sesame plants positively correlated to AM colonization (Chitarra et al. 2016). This might be the reason for increased AM colonization and AM spore in the experiment. Gholinezhad and Darvishzadeh (2019) also reported an increase in sesame protein and oil content when inoculated by *Funneliformis mosseae* and *Glomus intraradices*. Harikumar (2017) also confirmed an increased in yield and oil content, up to 50% in Sesame when inoculated by AMF. Work done by these researchers and many others corresponds to our findings. Moreover, under stress conditions, the symbiotic fungi like AMF thrive and provide extra nutrients to the roots, producing effects such as development and higher yields. AMP can protect plants from rapidly changing climates. As a result, more studies are required to understand the AMF role in increasing crop quality and crop plants' productivity (Ahanger et al., 2014, Hameed et al., 2014, Kumar et al., 2015, Latef et al., 2016, Malhi et al., 2021). The present assessment gives a current impression of the numerous nutrient requirements of AMF and their effects on different plant growth stages, including their crucial importance to oilseed crops like sesame, thereby demonstrating the significance of these dependencies on AMF.

## 6 Conclusion

*Pseudomonas* spp. and AMF are important microbial bioinoculants that have been shown to promote plant growth, yield and protect plants from pathogens and insects. These inocula help plants to attain healthy growth and high yield, even under stress condition. AMF and PSB can stimulate strigolactones which are iron-chelating agent important for plant growth hormones. It is very well acknowledged that AMF and PSB facilitates nutrient accumulation and is also found in the experiment. Future work will address that by application of bioinoculants, the use of synthetic fertilizers can be minimized with a view to maximize the yield. Overall, the amalgamation of AMF and PSB is the best treatment for attaining better growth and increased water and mineral absorption. Several essential components of the cellular and metabolic processes are maintained by minerals. Sesame biofortification using AMF would be a long-safe way to win over mineral deficiency and fight hidden hunger.

## Declaration of Competing Interest

The authors declare that they have no known competing financial interests or personal relationships that could have appeared to influence the work reported in this paper.

## References

[b0005] Ahanger M.A., Hashem A., Abd-Allah E.F., Ahmad P. (2014). Arbuscular mycorrhiza in crop improvement under environmental stress. Emerging Technologies and Management of Crop Stress Tolerance.

[b0010] Ambrosini A., de Souza R., Passaglia L.M.P. (2016). Ecological role of bacterial inoculants and their potential impact on soil microbial diversity. Plant Soil.

[b0015] Anokhina T.O., Siunova T.V., Sizova O.I., Kochetkov V.V., Boronin A.M. (2018). Strain of *pseudomonas fluorescens* for protecting plants from phytopathogenic fungi and bacteria and stimulating plant growth. Russian Patent, RU.

[b0020] AOCS (1997) Official methods and recommended practice of AOCS. The American Oil Chemist’s Society. Washington DC, 5th Edn.

[b0025] Arif M.S., Riaz M., Shahzad S.M., Yasmeen T., Akhtar M.J., Riaz M.A., Jassey V.E.J., Bragazza L., Buttler A. (2016). Associative interplay of plant growth promoting rhizobacteria (*Pseudomonas aeruginosa* QS40) with nitrogen fertilizers improves sunflower (*Helianthus annuus* L.) productivity and fertility of aridisol. Appl. Soil Ecol..

[b0030] Azooz MM, Ahmad P, 2013. Role of bio-fertilizers in crop improvement. Crop Improvement: New Approaches and Modern Techniques 189.

[b0035] Balestrini R., Chitarra W., Antoniou C., Ruocco M., Fotopoulos V. (2018). Improvement of plant performance under water deficit with the employment of biological and chemical priming agents. The Journal of Agricultural Science.

[b0040] Bheda K, 2019, India Sesame Crop 2019/20: Update & Outlook. World Sesame Convention 18-20 August, Istanbul.

[b0045] Bona E., Cantamessa S., Massa N., Manassero P., Marsano F., Copetta A., Lingua G., D’Agostino G., Gamalero E., Berta G. (2017). Arbuscular mycorrhizal fungi and plant growth-promoting pseudomonads improve yield, quality and nutritional value of tomato: A field study. Mycorrhiza.

[b0050] Bonfante P., Genre A. (2015). Arbuscular mycorrhizal dialogues: do you speak ‘plantish’ or ‘fungish’?. Trends Plant Sci..

[b0055] Bradford M.M. (1976). A rapid and sensitive method for quantitation of microgram quantities of protein utilizing the principle of protein-dye binding. Ann. Clin. Biochem..

[b0060] Cavagnaro T.R., Bender S.F., Asghari H.R., van der Heijden M.G.A. (2015). The role of arbuscular mycorrhizas in reducing soil nutrient loss. Trends Plant Sci..

[b0065] Cheema J., Yadav K., Sharma N., Saini I., Aggarwal A. (2016). Nutritional Quality Characteristics of Different Wild and Underutilized Fruits of Terai Region, Uttarakhand (India). Int. J. Fruit Sci..

[b0070] Chen M., Arato M., Borghi L., Nouri E., Reinhardt D. (2018). Beneficial Services of Arbuscular Mycorrhizal Fungi – From Ecology to Application. Front. Plant Sci..

[b0075] Chitarra W., Pagliarani C., Maserti B., Lumini E., Siciliano I., Cascone P., Schubert A., Gambino G., Balestrini R., Guerrieri E. (2016). Insights on the impact of arbuscular mycorrhizal symbiosis on tomato tolerance to water stress. Plant Physiol..

[b0080] Estefan G, Sommer R, Ryan J, 2013. Methods of Soil, Plant, and Water Analysis: A Manual for the West Asia and North; International Center for Agricultural Research in the Dry Area (ICARDA): Beirut, Lebanon,

[b0085] Estefan G, Sommer R, Ryan J, 2013. ‘Methods of Soil, Plant, and Water Analysis: A Manual for the West Asia and North.’ International Center for Agricultural Research in the Dry Area (ICARDA): Beirut, Lebanon

[b0090] FAOSTAT (2017) FAO Statistical data, Food and Agriculture Organization of the United Nations, Rome, http://www.fao.org/faostat/en/#data/QC (accessed on 11 December 2020).

[b0095] Gholinezhad E., Darvishzadeh R. (2019). Path analysis for seed yield in sesame (*Sesamum indicum* L.) inoculated/non-inoculated with mycorrhizal fungi under drought stress. Iranian J. Genet. Plant Breed..

[b0100] Graham S, Lam WVKY Merckx V, 2017. Plastomes on the edge: the evolutionary breakdown of mycoheterotroph plastid genomes. New Phytologist 214 48-55. 10.1111/nph.14398 28067952

[b0105] Habibullah M., Masjuki H.H., Kalam M.A., Rahman S.M.A., Mofijur M., Mobarak H.M., Ashraful A.M. (2015). Potential of biodiesel as a renewable energy source in Bangladesh. Renew. Sustain. Energy Rev..

[b0110] Harikumar V.S. (2017). Biometric parameters of field grown sesame influenced by arbuscular mycorrhizal inoculation, rock phosphate fertilization and irrigation. Trop. Subtrop. Agroecosyst..

[b0115] Hassan A.B., Mahmoud N.S., Elmamoun K., Adiamo O.Q., Mohamed A., Isam A. (2017). Effects of gamma irradiation on the protein characteristics and functional properties of sesame (*Sesamum indicum* L.) seeds. Radiat. Phys. Chem..

[b0120] Hameed A, Dilfuza E, Abd-Allah EF, Hashem A, Kumar A, Ahmad P, 2014. Salinity stress and arbuscular mycorrhizal symbiosis in plants, in: Use of Microbes for the Alleviation of Soil Stresses, Volume 1. Springer, pp. 139-159.

[b0125] Hashem A, Kumar A, Al-Dbass AM, Alqarawi AA, Al-Arjani A-BF, Singh G, Farooq M, Abd_Allah EF, 2019. Arbuscular mycorrhizal fungi and biochar improves drought tolerance in chickpea. Saudi Journal of Biological Sciences 26 614-624. 10.1016/j.sjbs.2018.11.005 PMC6408710 30899180

[b0130] Hwang H.J., Cheigh C.I., Chung M.S. (2017). Construction of a pilot-scale continuous-flow intense pulsed light system and its efficacy in sterilizing sesame seeds. Innovat. Food Sci. Emerg. Technol..

[b0135] Jia-Dong H., Tao D., Hui-Hui W., Ying-Ning Z., Qiang-Sheng W., Kamil K. (2019). Mycorrhizas induce diverse responses of root TIP aquaporin gene expression to drought stress in trifoliate orange. Sci. Hortic..

[b0140] Johri A.K., Oelmüller R., Dua M., Yadav V., Kumar M., Tuteja N., Varma A., Bonfante P., Persson B.L., Stroud R.M. (2015). Fungal association and utilization of phosphate by plants: Success, limitations, and future prospects. Front. Microbiol..

[b0145] Joshi A.U., Liu C., Sathe S.K. (2015). Functional properties of select seed flours. LWT – J. Food Sci. Technol..

[b0150] Kalita P.J., Ram R.M. (2019). Industrial Applications of *Pseudomonas fluorescens*: A Patent Survey.

[b0155] Kaushik P, Sandhu OS, Brar NS, Kumar V, Malhi GS, Harikesh and Saini I. (2020). Soil Metagenomics: Prospects and Challenges [Online First]. In ‘Mycorrhizal Fungi - Utilization in Agriculture and Industry’. pp. 1-18. (IntechOpen Ltd.: London, UK)

[b0160] Konieczny A., Kowalska I. (2017). Arbuscular mycorrhiza – partner in communication. Acta Scientiarum Polonorum Hortorum Cultus.

[b0165] Kumar A., Dames J.F., Gupta A., Sharma S., Gilbert J.A., Ahmad P. (2015). Current developments in arbuscular mycorrhizal fungi research and its role in salinity stress alleviation: a biotechnological perspective. Crit. Rev. Biotechnol..

[b0170] Kurt C. (2018). Iron and Zinc content some turkish sesame (*Sesamum indicum* L.) accessions. Int. J. Agri. Environ. Res..

[b0175] Lanfranco L., Fiorilli V., Gutjahr C. (2018). Partner communication and role of nutrients in the arbuscular mycorrhizal symbiosis. New Phytol..

[b0180] Latef AAHA, Hashem A, Rasool S, Abd_Allah EF, Alqarawi AA, Egamberdieva D, Jan S, Anjum NA, Ahmad P (2016) Arbuscular mycorrhizal symbiosis and abiotic stress in plants: a review. Journal of Plant Biology 59 407-426.

[b0185] Liu G., Pfeifer W., Francisco J., Emonet R.D., Stirnemann A., Gubeli M.C. (2018). Changes in the allocation of endogenous strigolactone improve plant biomass production on phosphate-poor soils. New Phytol..

[b0190] Malhi G.S., Kaur M., Kaushik P., Alyemeni M.N., Alsahli A.A., Ahmad P. (2021). Arbuscular mycorrhiza in combating abiotic stresses in vegetables: An eco-friendly approach. Saudi J. Biolog. Sci..

[b0195] Mitchell G.A., Bingham F.T., Yermanos D.M. (1974). Growth, mineral composition and seed characteristics of sesame as affected by nitrogen, phosphorus and potassium nutrition. Soil Sci. Soc. Am. J..

[b0205] Myint D., Gilani S.A., Kawase M., Watanabe K.N. (2020). Sesame (*Sesamum indicum* L.) Production through improved technology: An Overview of Production, Challenges, and Opportunities in Myanmar Sustainability. Sustainability.

[b0210] Park J., Lee Y., Martinoia E., Geisler M. (2017). Plant hormone transporters: What we know and what we would like to know. BMC Biol..

[b0215] Rashid M.I., Mujawar L.H., Shahzad T., Almeelbi T., Ismail I.M., Oves M. (2016). Bacteria and fungi can contribute to nutrients bioavailability and aggregate formation in degraded soils. Microbiol. Res..

[b0220] Rouphael Y., Franken P., Schneider C., Schwarz D., Giovannetti M., Agnolucci M. (2015). Arbuscular mycorrhizal fungi act as biostimulants in horticultural crops. Sci. Hortic..

[b0225] Saini I., Aggarwal A., Kaushik P. (2019). Inoculation with mycorrhizal fungi and other microbes to improve the morpho-physiological and floral traits of *Gazania rigens* (L.) Gaertn. Agriculture.

[b0235] Saini I., Aggarwal A. and Kaushik P., 2019b. Influence of biostimulants on important traits of Zinnia elegans Jacq. under open field conditions. International Journal of Agronomy 2019 3082967.

[b0240] Saini I., Chauhan J., Kaushik P. (2020). Medicinal value of domiciliary ornamental plants of the Asteraceae family. J. Young Pharm..

[b0245] Saini I., Himanshi R.K., Gill N., Sandhu K., Bisht N., Kumar T., Kaushik P. (2020). Significance of Arbuscular Mycorrhizal Fungi for *Acacia*: A Review. Pak. J. Biol. Sci..

[b0255] Saini I., Yadav V.K., Kaushik P. (2020). Effect of superphosphate, urea and bioinoculants on Zi*nnia elegans* Jacq. Indian J. Exp. Biol..

[b0260] Song X., Liu M., Wu D., Griffiths B.S., Jiao J., Li H., Hu F. (2015). Interaction matters: Synergy between vermicompost and PGPR agents improves soil quality, crop quality and crop yield in the field. Appl. Soil Ecol..

[b0265] Spatafora J.W., Chang Y., Benny G.L., Lazarus K., Smith M.E., Berbee M.L. (2016). A phylum-level phylogenetic classification of zygomycete fungi based on genome-scale data. Mycologia.

[b0270] Wang M., Jiang P. (2015). Colonization and diversity of am fungi by morphological analysis on medicinal plants in Southeast China. Sci. World J..

